# MSC-derived exosomes carrying a cocktail of exogenous interfering RNAs an unprecedented therapy in era of COVID-19 outbreak

**DOI:** 10.1186/s12967-021-02840-3

**Published:** 2021-04-22

**Authors:** Monire Jamalkhah, Yasaman Asaadi, Mohammadreza Azangou-Khyavy, Javad Khanali, Masoud Soleimani, Jafar Kiani, Ehsan Arefian

**Affiliations:** 1 Department of Biotechnology, College of Science, University of Tehran, Tehran, Iran; 2 School of Medicine, Shahid Beheshti University of Medical Sciences, Tehran, Iran; 3 Department of Tissue Engineering and Applied Cell Sciences, School of Advanced Technologies in Medicine, Shahid Beheshti University of Medical Sciences, Tehran, Iran; 4 Department of Molecular Medicine, School of Advanced Technologies in Medicine, Iran University of Medical Sciences, Tehran, Iran; 5 Department of Microbiology, School of Biology, College of Science, University of Tehran, Tehran, Iran

**Keywords:** Mesenchymal stem cells, COVID-19, SARS-CoV-2, Exosomes, RNA interference, COVID-19 therapy

## Abstract

**Background:**

The onset of the SARS-CoV-2 pandemic has resulted in ever-increasing casualties worldwide, and after 15 months, standard therapeutic regimens are yet to be discovered.

**Main body:**

Due to the regenerative and immunomodulatory function of MSCs, they can serve as a suitable therapeutic option in alleviating major COVID-19 complications like acute respiratory distress syndrome. However, the superior properties of their cognate exosomes as a cell-free product make them preferable in the clinic. Herein, we discuss the current clinical status of these novel therapeutic strategies in COVID-19 treatment. We then delve into the potential of interfering RNAs incorporation as COVID-19 gene therapy and introduce targets involved in SARS-CoV-2 pathogenesis. Further, we present miRNAs and siRNAs candidates with promising results in targeting the mentioned targets.

**Conclusion:**

Finally, we present a therapeutic platform of mesenchymal stem cell-derived exosomes equipped with exogenous iRNAs, that can be employed as a novel therapeutic modality in COVID-19 management aiming to prevent further viral spread within the lung, hinder the virus life cycle and pathogenesis such as immune suppression, and ultimately, enhance the antiviral immune response.

## Background

As of December 2020, roughly 67 million confirmed cases with severe acute respiratory syndrome coronavirus 2 (SRAS-CoV-2) infection had been reported, with over a million and a half demises [1]. In response to the SARS-CoV-2 outbreak, many therapeutic approaches have been proposed and clinically evaluated to reduce the Coronavirus Disease 2019 (COVID-19) mortality rate. However, there is no unanimously approved product in the global market as COVID-19 therapy for SARS-Cov-2 positive patients [1]. Hence, developing new therapeutics, particularly advanced therapeutic platforms, is still enduring.

Although gene transfer-based approaches have been singularly exploited in vaccine design and multiple candidates are now under clinical evaluation [2], except DeltaRex-G, no gene therapy has been clinically tested for COVID-19 treatment (NCT04378244). Considering the extensive research aimed at developing short interfering RNAs for therapeutic purposes, RNA interference can serve as a genetic treatment approach for SARS-CoV-2 critically ill cases. Due to their natural characteristics, exosomes are considered suitable carriers for interfering RNA (iRNA) delivery.

Exosomes collected from several cell types have shown promise in inducing remission in virally infected patients, especially SARS-CoV-2 positive individuals [3, 4]. Multiple clinical trials are now assessing the administration of mesenchymal stem cell (MSC)-derived exosomes in critically ill COVID-19 patients [4].

This article reviews the current status of exosome-based therapies, particularly those derived from MSCs and their promise as genetic material-delivery vectors. The MSCs’ immune-modulatory and regenerative capabilities in alleviating pulmonary complications, specifically COVID-19, are then elaborated, serving as a rationale for their assignment as the exosome source. Afterward, we delve into the ongoing clinical studies on the administration of exosomes on COVID-19 treatment. Thereafter, the promise of RNA interference (RNAi) -based gene therapy for COVID-19 is explained.

Then, the potential interfering RNA candidates and their cognate targets are introduced in four classes, pro-viral microRNAs (miRNAs), the viral genes themselves, host genes mediating the virus entry and replication, and those of hosts playing roles in the induction of hyper-inflammation.

We culminate by depicting a pipeline for the administration of MSC-derived secretomes carrying a cocktail of the mentioned iRNAs as a novel therapeutic approach for COVID-19 patients in critical status.

## Main text

### Immunomodulatory and regenerative capacity of MSCs

Upon its entry into the lung through respiration, SARS-CoV-2 primarily invades and destroys pulmonary epithelial cells. The released viral molecular structures are then recognized via pattern recognition receptors (PRRs) on lung-resident innate immune cells, including dendritic cells and macrophages. The local immune response is then triggered, and inflammatory cytokines and chemokines are produced, attracting other immune cells, including T lymphocytes and monocytes. Under normal circumstances, the subsequent anti-viral immune response wipes out the virus with minimal damage before its extensive spread throughout the body. However, the aberrant hyper-inflammatory response in some individuals results in a sudden release of an excessive amount of pro-inflammatory cytokines, a process known as “cytokine storm” [5–7]. Cumulative reports correlate the severity of COVID-19 with the excessively-heightened level of pro-inflammatory mediators including interleukin 1 (IL-1), interleukin 6 (IL-6), tumor necrosis factor-alpha (TNF-α), alongside multiple agents, lists of which reported elsewhere [8–11]. As a result of cytokine storm, blood circulating immune cells, including neutrophils and T lymphocytes, are outrageously recruited into the lung, leading to significant tissue damage, and consequently, lung injury. Lung injury may progress into acute respiratory distress syndrome (ARDS), which is the leading cause of morbidity among COVID-19 patients [12].

MSCs are well known for their profound performance in immunomodulation and tissue repair when encountering a highly-inflamed milieu, particularly in the lung. These cells exert their immunomodulatory and reparative impacts either via direct cell–cell interaction or through the paracrine release of the underlying mediators, including the cell’s migratory elements, immune regulatory agents, antiapoptotic factors, and angiogenic mediators [13–15].

They shift the immune system status from inflammation toward regulatory mode by suppressing T lymphocytes’ proliferation and converting the balance between Th1 inflammatory cells and T regulatory cells toward the latter [16, 17]. They also induce the conversion of pro-inflammatory M1 macrophages to anti-inflammatory M2 ones, which in turn results in reduced neutrophil infiltration into the lung [18, 19]. MSCs are also reported to inhibit dendritic cell maturation and activation and also prevent natural killer cell function and proliferation [74], and prevent dendritic cell (DC) maturation and activation [20, 21].

Following modulating the immune response within the lung, they instigate the regeneration of the injured tissues through reversing lung dysfunction and halting pulmonary fibrosis [22]. These dual beneficial therapeutic mechanisms paved MSCs’ way into multiple clinical studies on COVID-19 therapy [23].

Although the safety and efficacy of MSC-based treatment are demonstrated by a handful of studies, the possibility of SARS-Cov-2 infection on MSCs was unknown. Recently a study conducted in china illustrates that the ACE2 and TMPRSS2—the two vital receptors for viral entry—are not expressed on MSC cells, and injection of MSC has no role in inducing infection of other cell types [24]. The result of this study revealed the safety of MSC-based therapy for COVID-19 patients.

Despite its tremendous benefits, utilizing MSCs as immunomodulatory and regenerative agents is not devoid of limitations, particularly when administered through the IV route [25]. For example, these cells might be entrapped at the capillary level and can be almost cleared from the circulation, with a small proportion of them surviving on their way to the target site [26, 27]. Therefore, for efficient trafficking of the cells to the target site, a sufficient cell number needs to be administered and monitored, which can be a resource-consuming process [28]. Moreover, MSCs express tissue factor (TF/CD142) that raises the concern of thromboembolic events [25].

### Exosomes as therapeutic agents

After almost three decades since the first report [29], exosomes are now recognized as vital mediators of cell–cell communication [3, 29–31]. They are also key players in fundamental cell biology and pathologies, including cancer [32] and cardiovascular diseases [33]. Exosomes are extracellular vesicles of endocytic origin that are secreted by almost every cell type and typically range in 30–100 nm size [30]. They carry macromolecules, including lipids, proteins, and nucleic acids (mainly RNA), and their composition depends on their parent cell [3, 4, 29–31].

Secreted exosomes containing biologically active macromolecules can deliver their cargo to the target cell by two distinct mechanisms. First, following the selective binding to cell surface receptors, exosomes are thought to transduce specific intracellular signaling, thereby inducing physiological changes in recipient cells [34]. The second mechanism is the direct transfer of intra-exosomal content such as mRNA and miRNAs into the recipient cells by fusion with the cell membrane [35].

From the pharmaceutical aspect, exosomes demonstrate therapeutic potential when utilized in their native form. For instance, exosomes originated from stimulated platelets have demonstrated superior efficiency in occlusive thrombosis suppression [36]. Furthermore, MCS-derived secretomes, which will be discussed later, display a wide range of capabilities as native extracellular vesicles, including regenerative function in skin, muscle, cardiac and skeletal injuries [37]. However, as illustrated later, exosomes can be manipulated as nanocarriers to deliver various medicinal cargo, including non-coding RNAs (ncRNAs), to the cells of interest as well [38].

#### Clinical studies on exosome-based COVID-19 therapy

The present pandemic renewed many researchers' interest in the applicability of exosomes as an effective and safe therapeutics for combating COVID-19 associated diseases. As of November 2020, seven clinical trials have been submitted on clinicaltrials.gov to evaluate the safety and/or efficacy of exosome-based therapeutic regimens on SARS-CoV-2 positive patients (Table 1).

**Table 1:** Ongoing clinical trials on exosome therapy in COVID-19 patients

NCT number	Locations	Cell source	Administration route	Frequency of administration	Dosage	Criteria	Age	Number of patients	Phase	Result	Status
4276987	China	Adipose mesenchymal stem cells	Aerosol inhalation	Daily, day 1 to day 5	2 × 10^8^/3 mL	Individuals with severe COVID-19 symptoms	18 to 75 years old	30	Phase 1	Not yet publicly available	Completed
4384445	USA	Human amniotic fluid (HAF)	Intravenous injection	Day 0, day 4 and day 8	2–5 × 10^11^/mL	Individuals with moderately to severe COVID-19 symptoms	18 years and older	20	Phase 1&2	Not yet	Recruiting
4602442	Russia	N/A	Aerosol inhalation	Twice a day for 10 days	N/A	Hospitalized COVID-19 positive patients	18 to 65 years old	90	Phase 2	Not yet	Enrolling by invitation
4389385	Turkey	COVID-19 specific T-cells	Aerosol inhalation	Daily, day 1 to day 5	2 × 10^8^/3 mL	COVID-19 positive patients with Early Stage NCV Pneumonia	18 to 75 years old	60	Phase 1&2	Not yet	Active, not recruiting
4493242	USA	Bone marrow	Intravenous injection	N/A	N/A	COVID-19 patients with moderate-to-severe ARDS	18 to 85 years old	60	Phase 2	Not yet	Not yet recruiting

##### MSC-derived exosome therapy in COVID-19

MSC-derived exosomes can be considered as an alternative since they are repeatedly proven to exert similar immune-modulatory and regenerative impacts under distinct circumstances, including hyper-inflammatory situations during pulmonary complications [39–45]. Despite the potency of MSCs for COVID-19 therapy, MSC-derived exosomes are a better option in the clinic in comparison to their cellular counterparts. While they lead to the same result, MSC-derived exosomes as a cell-free product are more stable, easier to store, and less immunogenic [37], making it a superb substitute as a treatment for several diseases, including lung injury [46]. Furthermore, the cost-effectivity of these natural products makes them a superior therapeutic option for pandemics. Especially in underdeveloped countries, that lack of proper facilities hampers the utilization of any cell-based therapies, whereas the easier delivery of exosomes as freeze-dried powder augment their accessibility in these regions [47, 48]. As another advantage over cell-based therapies, exosomes can also be administrated non-invasively through inhalation [49], which lowers the dosage and prevents the costs and side effects accompanying IV injection.

As the first of its kind, in a pilot phase I study in Ruijin Hospital, China, allogenic adipose MSC-derived exosomes (MSCs-Exo) were administrated to severe patients afflicted with SARS-CoV-2 pneumonia through aerosol inhalation (NCT04276987). Although the study was reportedly completed in July 2020, the results are yet to be published. In another ongoing parallel clinical study on healthy volunteers, Rujin Hospital evaluates the safety and tolerance and determines the clinical dose reference for the aerosol inhalation of the exosomes mentioned above (NCT04313647).

In phase I prospective nonrandomized open-label cohort study during April 2020, Direct Biologics demonstrated the safety and efficacy of its product ExoFlo™. This product is made of allogeneic bone marrow mesenchymal stem cells-derived exosomes and has been tested on 24 severe COVID-19 patients with moderate-to-severe ARDS. A single intravenous injection of ExoFlo displayed no adverse event, and a survival rate of 83% was observed with the restoration of oxygenation, significant improvements in absolute neutrophil count and lymphopenia, and reduction in acute phase reactants including C-reactive protein, ferritin, and d-dimer [4]. Consequently, a phase II multicenter randomized double-blinded placebo-controlled trial study (EXIT COVID-19) is planned to assess ExoFlo’s potential in treating moderate-to-severe ARDS in COVID-19 patients (NCT04493242).

In July 2020, Russia launched a study to assess the efficacy of aerosol inhalation of the exosomes in treating severe patients hospitalized with novel coronavirus pneumonia, joining the race toward having MSC-derived secretome designated against cytokine release syndrome-mediated ARDS (NCT04491240).

However, it is worth noting that there are hurdles that need to be considered in the application of MSC-derived exosome therapy in the COVID-19. These hurdles include developing established methods of isolation, loading, real-time monitoring of trafficking, and potential off-target effects of the exosomes. For example, the targeting efficacy of theses delivery tools can be enhanced by attaching novel ligands specific to the target tissue [50]. Moreover, similar to the MSC administration, the pro-coagulant ingredients of the exosomes are a major aspect that needs to be focused on. COVID-19 patients are at the risk of hypercoagulable state (e.g., disseminated intravascular coagulation (DIC) and thrombo-embolism), and these adverse effects have been reported following the administration of some MSC products [25, 51]. Hence, in the clinical settings, the coagulative state of patients needs to be monitored, and preventive measures have to be adopted. Finally, due to the ongoing pandemic’s complexities, along with growing global demand, it is crucial yet challenging to develop robust logistics to provide sufficient and efficient MSCs and exosomes as their products in a consistent manner.

##### Non-MSC-derived exosome therapy in COVID-19

Extracellular vesicles derived from other cells have also paved their way to clinical studies for COVID-19 treatment. CSTC-Exo is a product based on the exosomes derived from virus-specific T lymphocytes, which are activated and expanded in vitro by their exposure to viral peptide fragments in the presence of activating and co-stimulatory signals. These T cells-secreted exosomes carry immune mediators, and furthermore, they can serve as off-the-shelf therapeutics in contrast with the virus-specific T cell-based immunotherapy, which is mostly HLA-restricted. In a single-arm open-labeled combined interventional (phase I/II trials) clinical trial in TC Erciyes University, Turkey, CSTC-Exo is being administrated to patients at early stages of SARS-CoV-2-related pulmonary disease. This medication is being delivered via a metered-dose inhaler to assess its potential in halting the disease progression (NCT04389385).

Zofin (Organicell Flow) is another non-MSC-derived exosome-based medication developed by Organicell Regenerative Medicine and is under evaluation for its safety and potential efficacy profile in a phase1/2 clinical trial for the treatment of COVID-related moderate to severe acute respiratory syndrome. Zofin is an acellular, minimally manipulated product derived from human amniotic fluid (HAF) and contains various anti-inflammatory agents such as commonly known miRNAs (NCT04384445).

### RNAi as a gene therapy agent

ncRNAs are post-translational gene silencers and guide the mechanism of sequence-specific gene regulation through a process called RNA interference (RNAi). There are two types of RNAi mediators in this process: small interfering RNAs (siRNAs) and miRNAs.

siRNAs are a part of antiviral immunity that target viral genes and silence their expression. siRNA therapeutic potentials were recently (2018) confirmed after the FDA-approval of the first siRNA-based drug (i.e., Patisiran by Alnylam) for the treatment of nerve damage in hereditary transthyretin-mediated amyloidosis (hATTR) in adults [52]. Antiviral siRNA-based therapeutics have also entered clinical trials against various viral infections, including HIV, Ebolavirus, and RSV, illustrating efficacy in inhibiting the replication of various viral pathogens despite distinct mechanisms exploit to evade host immunity [53–55].

MicroRNAs, as another class of RNAi, can regulate post-transcriptional-level gene expression in a broader range [56]. In viral infections, the host miRNA expression plays a major role in controlling the replication of the virus by direct binding to the viral genome [57] and mediating T cells and antiviral effector functions [58]. miR-32, the first-ever miRNA targeting viral RNA, binds to the retrovirus PFV-1 transcripts and diminishes the virus replication [58].

Two modalities are mostly recruited concerning miRNA-based therapies, miRNA mimics, and anti-miRNA oligonucleotides. miRNA mimics delivery serves to restore a given miRNA concentration, which had been suppressed as a part of the pathology of the disease. Conversely, anti-miRNA oligonucleotides target perilously overexpressed miRNAs. Both strategies are being vastly assessed in clinical trials for various complications [59, 60].

Since RNAi-based therapeutics have demonstrated promising outcomes in treating various pulmonary diseases [61, 62], including earlier SARS virus [63], RNAi-based drugs for SARS-CoV-2 could emerge as a potential treatment for hospitalized patients.

#### Choosing the right cocktail of iRNAs for COVID-19 therapy

SARS-CoV-2 entry mechanisms into the lung epithelium have long been established to be mediated by binding of the virus spike (S) protein to the angiotensin-converting enzyme 2 (ACE2) receptor and the subsequent S protein priming via transmembrane serine protease 2 (TMPRSS2) processing [64, 65]. Upon cell entry, SARS-CoV-2 hijacks multiple cellular pathways and machinery to propagate and damp immune response and ultimately debilitate the host’s survival upon the virus infection.

Intervention in the pathways involved in the virus pathophysiology can theoretically block the virus propagation and pathogenesis via targeting either the virus genes or the host genes harnessed by the virus, mostly the ones involved in the viral entry and replication and immune escape and the following hyper-inflammation induction.

**Fig. 1:**
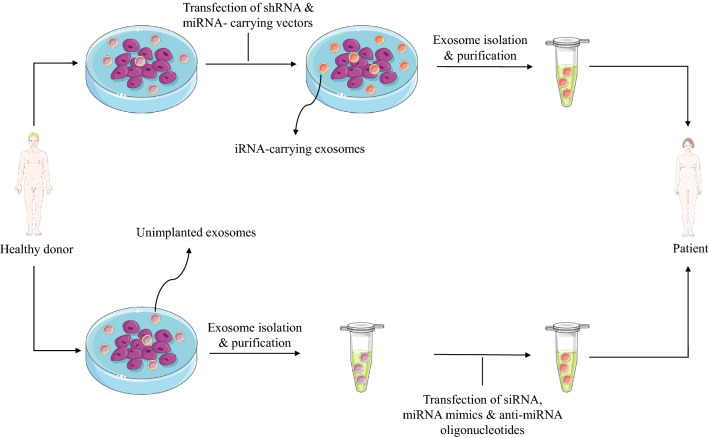
Potential targets for interfering RNAs in COVID-19. Targeting essential viral genes within the conserved regions of its genome hampers the virus’s cycle of life. As the virus-encoded miRNAs and host pro-viral miRNAs contribute to the virus’s pathogenesis, their hindrance via anti-miRNA oligonucleotides can disrupt the mentioned mechanisms. Human genes responsible for viral entry and the ones hijacked by the virus can also serve as promising iRNAs targets. Targeting various inflammatory genes associated with the SARS-CoV-2 clinical manifestations like ARDS can alleviate the COVID-19 respiratory complications

Multiple studies have unveiled RNAi candidates that target the virus transcripts and also the host mRNAs, genes of which take part in the virus pathogenesis. RNAi-dependent gene expression manipulation has also been repeatedly demonstrated to partly mediate the virus pathophysiology. Virus-originating miRNAs and the host cell’s upregulated miRNAs, which contribute to the virus replication cycle, can additionally serve as potential therapeutic targets (Fig. 1) [66].

##### Viral genes

SARS-CoV-2 genome has 14 open reading frames (ORFs) and encodes 27 proteins, of which four are structural, an envelope protein (E), Nucleocapsid protein (N), matrix protein (M), and spike protein (S). Fifteen non-structural proteins (NSPs) within the ORF1a and ORF1b regions are located at the 5′ end of the genome, and the 3′ end of the genome comprises the sequences pertaining to eight accessory proteins and structural proteins [67]. The regions of interest for iRNAs targeting should be highly conserved in terms of mutational rate and essential for the viral life cycle. N and E proteins-encoding genes and RNA-dependent RNA polymerase (RdRp) gene are highly conserved and encode elements that are indispensable for viral replication and spread; hence they can serve as appropriate iRNA candidates design [68–71].

Designing siRNAs is a versatile process, and multiple siRNA designing approaches exist and have been reviewed elsewhere [72]. Several groups have been developing siRNAs against conserved regions of the virus genome. Using computational analysis, Lin et al. introduced nine potential siRNAs against RdRp and N and other genes, but their efficacy is yet to be assessed experimentally [73]. In another study, of 78 siRNA candidates, eight were predicted to effectively target N and S genes [74]. Major pharmaceuticals have also initiated the development of siRNA-based therapy for COVID-19. Vir Biotechnology and Alnylam Pharmaceuticals have joined forces to assess 350 siRNAs designed against SARS-coronavirus genomes, including the conserved regions [75]. Throughout separate projects, OilX Pharmaceuticals and Sirnaomics are also exploring the potential of siRNAs targeting the virus’s crucial genes [76, 77].

When designing siRNAs, it is worth considering that following SARS-CoV-2 entry to the cell, the positive-sense, single-stranded genomic RNA is translated into viral polymerase proteins. Subsequently, the complementary negative-sense RNA is synthesized and the regions encoding the structural proteins, and some accessory proteins begin to serve as a template for viral mRNA transcription [78, 79]. Therefore, the RNAs encoding these proteins are present with higher copy numbers than those encoded by the ORF1a and ORF1b.

Accordingly, the virus’s various genome loci are differently present in the host and hence will be targeted disproportionately. Genes within the first 20 kb portion of the virus’s genome are present in two forms, positive and negative sense strands. This portion holds the sequences of ORF1a and ORF1b, and designing a siRNA against the sequence within these loci would theoretically lead to the viral genome double-targeting. With respect to the 10 kb of the genome at the 3′ end, the negative and positive sense strands alongside the viral mRNAs could be triple-targeted via both siRNA strands, yielding a higher viral propagation inhibition [80]. However, recently there has been evidence that targeting sequences within the 10 kb of the genome at 3′ end would decrease siRNA's drug efficacy. The reason could be that high amounts of sub-genomic replicates compete with genomic RNAs for binding to siRNAs and RNA-induced silencing complex (RISC) for subsequent cleavage. Therefore, the ORF1, which is contained solely in genomic RNA, was found to be the most effective target against the SARS-CoV-2 genome. However, this higher efficiency may also be due to better accessibility of ORF1 for RNAi machinery because of the ORF1’s secondary RNA structure or lower abundance of the nucleocapsid proteins [81]. The study also showed that during the RNAi targeting, the negative-sense genomic RNA remains untouched; however, we believe that this strand could also be targeted by changing siRNA strands’ thermodynamic stability at 5′ ends. In this unique situation, the lack of preference between the two siRNA strands gets Argonaute to load both of them into the RISC, which in turn leads to simultaneous targeting of target both negative and positive-sense viral genomic RNAs [82]. Further empirical evidence is needed to identify the best target site in the SARS-CoV-2 genome for RNAi machinery.

Human miRNAome has been exhaustively explored to select miRNAs with prospective potential against the virus genes. Of the human miRNA repertoire, seven miRNAs were predicted to target and inhibit SARS-CoV-2 genes, including N [83]. In a study by Liu et al., human hsa-miR-4661-3p was revealed to target the N gene, serving as a host antiviral response [84]. Adan et al. also identified 479 human miRNAs against various SARS-CoV-2 genes, including N, E, and RdRp [85]. In an attempt to differentiate the epigenetic regulation between various pathogenic coronaviruses, Khademul Islam et al. identified 106 host antiviral miRNAs against SARS-CoV-2, of which three had displayed experimental evidence of having antiviral roles during infections [86]. In an integrated sequence-based analysis of SARS-CoV2 genomes, nine miRNAs were identified to target the SARS-CoV-2 genome, of which six also had targets on human genes, including *IFNB* as well [87].

##### Pro-viral miRNAs

Numerous studies have discovered viral miRNAs and pro-viral human miRNAs contribution to virus pathogenesis, some of which shedding light on their ablation via anti-miRNA oligonucleotides. Anti-miRNA oligonucleotides are synthetic oligonucleotides neutralizing miRNAs of interest [88]. Computational analysis of SARS-CoV-2 genome predicted putative viral miRNAs against antiviral response-mediating genes, including human genes involved in pathways like EGF receptor signaling, apoptosis signaling, VEGF signaling, FGF receptor signaling [89]. Using the same approach, Liu and colleagues predicted 45 miRNAs on the virus genome, of which 40 targeted 3′ UTR of 73 human genes, mostly involved in immune response, and 11 targeted 5′ UTR of 13 genes, and many of them are engaged in cytoskeleton organization. This study further demonstrated that viral MR147-3p elevated TMPRSS2 expression in the gut. Several virus-encoded miRNAs were also found to target 5′ UTR of viral genes encoding structural proteins [84]. In a study by Adan et al., viral miRNA-like oligonucleotides were found to target 1367 human genes, resulting in nullifying the immune system’s impact and decreasing the host transcription rate to benefit viral gene expression, a phenomenon named “Host shutoff” [85]. Khademul Islam and colleagues disclosed 170 SARS-CoV-2 mature miRNAs with the potential to target host genes involved in host immune responses, such as autophagy, ErbB signaling, VEGF signaling, Wnt signaling, FGF receptor binding, T-cell-mediated immunity, mTOR signaling, TGF-beta signaling, TNF-alpha signaling, and MAPK signaling [86].

##### Host genes

The number of identified host genes and pathways involved in viral entry, replication, and pathogenesis is on the rise, and their targeting has been introduced as a therapeutic intervention for COVID-19. Numerous studies are evaluating their perturbation to identify proteins and pathways exhibiting antiviral impacts.

Viral entry is mediated via membrane-bound ACE2 protein binding on lung cells, and its presence on infected cell surface declines due to endocytosis with the viral particle, and this event participates in the disease’s pathogenesis. Although it may seem like an exciting candidate, its knockdown accompanies serious side effects [90, 91]. Nonetheless, inhibition of Type 1 Angiotensin II Receptor (ATR1), which is stimulated during the virus infection, is proven to ameliorate acute lung failure in mice models [92]. Furthermore, TMPRSS2 protein convertase (PC) and cathepsin B/L also contribute to virus entry, and their blockade has been proposed as a promising therapeutic strategy [65, 93, 94].

Furin, namely paired basic amino acid cleaving enzyme (PACE), is another PC and mediates the exposure of S protein binding and fusion domains and is indispensable for the virus entering the cell. Inhibition of furin may have a therapeutic potential via blocking viral entry in SARS-CoV-2 and other viruses that possess the furin cleavage domain. Furin protein inhibitors demonstrated promising outcomes in various pathogens disease models, including influenza A virus, Pseudomonas aeruginosa, and HIV. GM-CSF bi-shRNA furin plasmid (VP) carries two short hairpin RNAs (shRNAs) against furin is now under clinical evaluation for Ewing’s sarcoma and ovarian cancer and is proposed as a repurposing drug for inhibition of viral propagation and immune response promotion [95, 96].

In a genome-wide CRISPR-based screening assay, Wilen et al. identified Cathepsin L, a mediator of viral entry through endocytosis [97], the SWI/SNF chromatin remodeling complex, and SMAD3 protein, a member of the TGF-β signaling pathway, as novel pro-viral agents and their inhibition via small molecules demonstrated therapeutic potential [98]. Construction of the gene network expression revealed genes co-expressed with ACE2 and TMPRSS2 and presented ADK, DPP4, IL13RA2, HDAC8, and CD55 as potential therapeutic targets [99]. Krogan et al. also identified 66 druggable human proteins or host factors by constructing a protein–protein interaction map between the host and SARS-CoV-2 proteins [100].

The possibility of the iRNAs’ efficacy against the mentioned genes as COVID-19 therapy is yet to be assessed, and to our knowledge, only one study has been conducted from this perspective. Seven candidate miRNAs were revealed in a study by Ramakrishnan and colleagues to target host-encoded proteins in signaling pathways involved in receptor activation and host protein hijacking machinery during the pathogenesis of SARS-CoV-2 [101].

Anti-miRNA oligonucleotides can also be designed to target the host miRNAs that assist viral pathogenesis. SARS-CoV-2 infection-induced human miRNAs are found to downregulate multiple pathways in antiviral defense response, including different Toll-Like Receptors (TLRs) [86].

##### Inflammatory genes

One of the clinical manifestations of COVID-19 is viral-induced inflammation, leading to ARDS. This syndrome is preceded by a significant rise in inflammatory parameters, such as C-reactive protein (CRP) levels, serum ferritin, the erythrocyte sedimentation rate, and d-dimers as a result of pro-inflammatory cytokines increase [102].

Inflammation generally consists of four steps, stimuli recognition by PRRs, inflammatory pathways activation, the release of inflammation mediators, and recruitment of immune cells to the inflammation site. Upon binding damage-associated molecular patterns (DAMPs) and pathogen-associated molecular patterns (PAMPs) to PRRs like TLRs and nod like receptors (NLRs), transcription factors within multiple inflammatory pathways including nuclear factor kappa B (NF-κB), mitogen-activated protein kinase (MAPK), and the Janus kinase signal transducer and activator of transcription (JAK–STAT) pathways translocate into the nucleus and upregulate the expression of various inflammatory cytokines and chemokines. A handful of inflammatory elements such as CRPs, high mobility group box protein 1 (HMGB1), superoxide dismutase (SOD), glutathione peroxidase-1 (GPx), NADPH oxidases (NOX), inducible nitric oxide synthase (iNOS), and cyclooxygenase-2 (Cox-2) are released from the afflicted cells and promote inflammation through their binding to inflammatory receptors [103]. Myriad of inflammatory signaling cascades involved in pulmonary diseases have been characterized, some of which have proof-of-concept contribution to the SARS-CoV-2 pathogenesis.

Cox-2 synthesizes prostaglandins in response to cytokines and mediates inflammation and tissue damage. Its promoter contains regulatory response elements to NF-kB and IL-6, and SARS-CoV N protein has been previously shown to induce its expression [104, 105]. Although in a recent clinical trial, Cox-2 targeting non-steroidal anti-inflammatory drugs h demonstrated safety in treating COVID-19 patients [106], its natural expression in the kidney poses a major drawback for its systematic blockade, making its localized inhibition an optimal situation [96].

Despite lack of experience in iRNA-mediated Cox-2 silencing in COVID-19, its knockdown and subsequent inflammation modulation have been vastly investigated for other diseases, including cancer and hepatic fibrosis. In a comprehensive review by Espisni et al., lists of studies analyzing various siRNAs and miRNAs are provided [107, 108]. In another study, miR-146a is proven to specifically inhibit Cox-2 in lung epithelial cells [109].

Of the MAPK signaling groups, the p38 MAPK pathway is aberrantly upregulated during SARS-CoV-2 infection, leading to the production of pro-inflammatory cytokines such as IL-6, TNF-α, and interleukin 1 beta (IL-1β). Multiple clinical studies are evaluating p38 inhibitors for a variety of complications [110]. Numerous studies have assessed siRNA candidates for p38 down-regulation in various afflictions, including breast cancer, in an ischemia–reperfusion injury lung transplantation model, and more importantly, lung adenocarcinoma cancer and all have been proven to be efficient suppressors leading to ameliorated inflammation [111–113].

Several miRNAs have also demonstrated efficacy in downregulating p38 and can serve as therapeutic candidates. In an attempt to reveal the mechanisms of action of three antiviral miRNAs, miR-124, miR-24, and miR-744, p38 was identified as a ubiquitous antiviral target in multiple viral infections, including influenza and respiratory syncytial virus (RSV) infection [114]. In an early pulmonary fibrosis mouse model caused by ARDS, miR-200b/c overexpression was concomitant with the inhibition of p38 MAPK and TGF-β/smad3 signaling pathways and alleviation of ARDS [115]. In a rat model of chronic inflammation, the miR-16 carrying vector administration palliated the inflammation-induced pain by inhibiting p38 activation [116]. miR-375 is found to prevent myofibroblast trans-differentiation and collagen synthesis by blocking the p38, which is a crucial pathophysiological process in pulmonary fibrosis [117].

NF-kB has repeatedly been demonstrated to orchestrate inflammation and contribute to inflammation-consequent pulmonary complications, including ARDS [118]. NF-kB is an established transcription factor in SARS-CoV pathogenesis. It is activated in response to the virus elements, including N protein [119], and accumulating evidence is attributing the same feature to it in COVID-19 as well. The binding of DAMPs to TLRs and cytokine receptors triggers NF-kB. Its activation upregulates pro-inflammatory agents, including IL-1b, IL-6, and TNF-a, leading to complications such as cytokine release syndrome CRS and pro-inflammatory immune cell recruitment. In a feedback-positive looping manner, these cytokines induce further activation of NF-kB [120, 121].

As a significant regulator of numerous inflammatory cytokines and chemokines, targeting NF-kB transcription factors inhibits multiple pro-inflammatory cascades simultaneously, serving as a superior therapeutic candidate. Cumulating evidence pinpoint the potential of NF-kB suppression in coronavirus-mediated SARS treatment as NF-kB inhibition in SARS-CoV animal models increased its survival and decreased pro-inflammatory agents’ expression [122]. The preliminary results of the RECOVERY clinical trial (NCT04381936) also ratify the rationale of NF-kB inhibition, wherein Dexamethasone, a chemical with NF-kB suppression as its mechanism of action, resulted in a significant reduction in COVID-19 critically ill patients [123, 124].

A manifold of siRNAs is designed and proven to effectively downregulate NF-kB or members of NF-kB signaling pathway and subsequently reduce expression of the NF-kB-regulated genes associated with inflammatory pathways in various pulmonary settings, including sepsis-induced acute lung injury in mice models, lipopolysaccharide-induced acute lung injury in rat models, lung cancer cells [125–128].

A myriad of miRNAs has been discovered which down-regulate the NF-kB pathway in various organs and modalities. Several papers listed the major miRNAs with altered expression levels in cancer with an impact on this pathway, some of which can serve as therapeutic candidates [129, 130]. Concerning the lung complications, upregulation of miR-140-5p is shown to dampen inflammatory cytokine production in acute lung injury via targeting the TLR4/MyD88/NF-κB signaling pathway [131]. miR-23b cluster and miR-125a-5p are confirmed to silence multiple components of KRAS and NF-kB pathways hence suppressing lung tumorigenesis [132]. By regulating the NF-κB/MMP-9/VEGF pathway, MicroRNA-26b is shown to suppress metastasis in lung cancer [133]. miR-449a also suppresses invasion of lung cancer through blocking HMGB1-Mediated NF-κB Signaling Pathway [134].

### RNAi suppressors as a challenge of using RNAi against SARS-CoV-2

It has long been established that interfering RNA-mediated defense mechanism against viruses is mostly confined to fungi, invertebrates, and plants. However, animal viruses are also discovered to be subject to the host RNAi-mediated suppression. Hence, within the evolutionary arms race between host and viruses, viruses have also evolved ways to nullify RNAi-mediated cellular anti-viral defense. Viral proteins underlying these mechanisms are referred to as RNAi suppressors. Aside from their roles in the viral life cycle in various ways, these proteins also manipulate histone and DNA methyltransferases as the components of the host’s transcriptional gene-silencing mechanisms to dampen the cellular antiviral silencing mechanism [135]. Many mammalian viruses, such as HIV and Ebola, were found to encode RNAi-blocking proteins [136]. Such RNAi suppressors were also found in the SARS-CoV; one is derived from ORF7a, and the other is SARS-CoV’s structural nucleocapsid protein [137, 138]. Given the homology of the two viruses, it is most likely that the 7a and N protein act as RNA interference suppressors as well in the SARS-CoV-2 [139]. Karjee et al. also recommended that both the full RdRP and the spike protein may be candidate RNAi suppressors in the SARS-CoV-2 genome, based on the motifs shared in these proteins and a subset of common RNAi suppressors [136]. Such RNAi suppressors might limit the efficiency of using RNAi technology against the SARS-CoV-2. Thus, targeting them could be considered as a strategy against the virus.

### Exosomes-based gene therapy for nucleic acid delivery

Utilizing exosomes as a drug delivery system was proposed in 2011 [140], and it gained much attention so far because of the exosomes’ small size, the capability to escape the immune system, deformable cytoskeleton, similarity to cell membranes, and slightly negative zeta potential, which allows them to circulate in the body for a longer period of time [140]. Noncoding RNAs are highly-suitable cargo for exosomes, that can target specific pathways to diminish inflammation in various diseases, including lung injury [141].

Due to the regenerative functions of exosomes secreted from MSC, they are an ideal source of exosomes in a variety of diseases such as cardiac ischemia, liver fibrosis, and cerebrovascular diseases. The therapeutic effects of miRNAs delivered by MSC-derived exosomes have been demonstrated by a handful of studies as well [142, 143].

In COVID-19, similar to other infectious diseases, the immune cells utilize miRNA-carrying exosomes to target the infected cells’ viral RNA. Thus, further delivery of specifically-designed ncRNAs by MSC-derived exosomes can accelerate the combat against SARS-CoV-2 and induce tissue regeneration [144]. To assess the anti-infectivity capacity of iRNAs inside the host cells’ exosomes, Moon et al. have unveiled anti-SARS-CoV-2 miRNA-content of MSC-derived extracellular vesicles [144]. This study sheds light on the mechanism of action of MSC-derived exosome as a carrier for nucleic acid-based therapies in COVID-19 to some extent.

### Production of iRNA-carrying exosomes

**Fig. 2:**
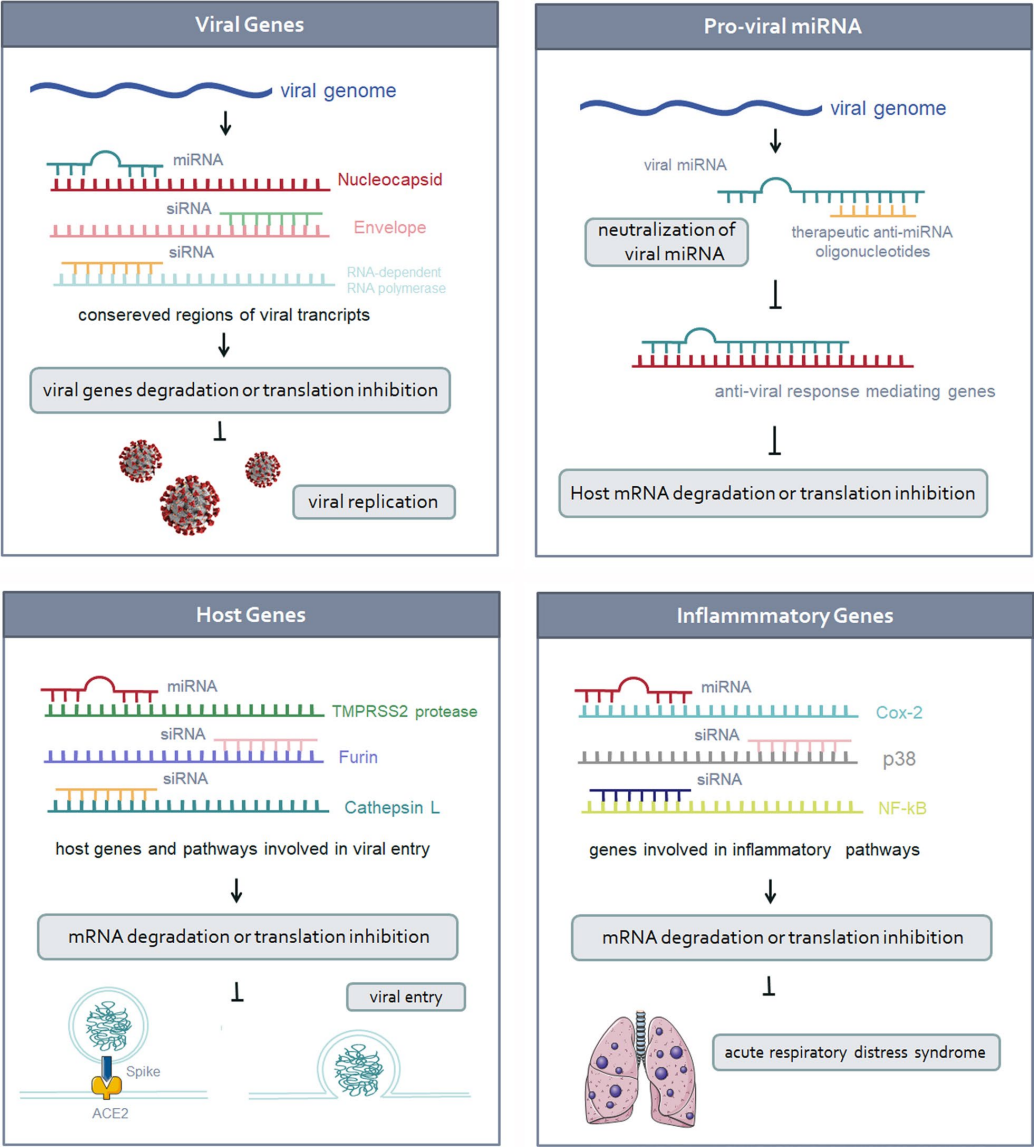
Pipeline of iRNA-carrying exosome production from MSCs. Therapeutic iRNA-carrying exosomes can be produced in two ways. The plasmid encoding the miRNA or/and shRNA of interest may be transferred into the MSCs, and the iRNA-containing exosomes will subsequently be harvested and enriched. Alternatively, synthesized miRNAs mimics or/and siRNAs or/and anti-miRNA oligonucleotides may be chemically inserted into the MSC-derived exosomes, and the resulting loaded exosomes will be then collected and isolated. The consequent exosomes of either way would then be administrated to the COVID-19 critically-ill patients

To combine the aforementioned anti-COVID-19 impact of MSC-derived exosomes with the iRNAs against SARS-CoV-2 pathogenesis, exosomes collected from MSCs should be loaded with the iRNAs of interest. MSCs of multiple sources can be used as exosome donors, including the umbilical cord, bone marrow, and adipose tissue [145]. Mainly, exosomes can be loaded with small RNAs either by direct insertion of the nucleic acids into them or by their collection from genetically-modified MSCs (Fig. 2).

When synthesized exogenously, siRNAs, miRNA mimics, and anti-miRNA oligonucleotides can be transferred into the exosomes via electroporation, lipofection, sonication, calcium chloride, co-incubation, or Saponin permeabilization [146, 147]. Multiple studies have reported the successful delivery of exogenous iRNAs into the MSC-derived exosomes and observed the expected functionality [148–151].

Alternatively, it is established that increasing the concentration of iRNAs in the cytosol of the cell is concomitant with their heightened copy number in exosomes [152]. In this regard, MSCs can be manipulated to express shRNA or miRNA of interest via transfection or transduction. The released exosomes can be isolated following their verification regarding the presence of the desired small RNAs. This methodology has demonstrated applicability in a handful of reports [153–156].

## Conclusion

Multiple clinical trials are assessing the efficacy of MSCs and MSC-derived exosomes in alleviating COVID-19 manifestations in critically-ill patients. Enrichment of MSC-derived exosomes carrying exogenous iRNAs for COVID-19 therapy serves as an unprecedented strategy and is yet to be exploited in clinical settings. The right cocktail of iRNAs would not only impede viral propagation, inflammation induction, and immune escape in already-infected cells but also can obstruct the viral particles’ entrance to the un-infected cells and the virus’s further spread within the lung tissue.

## Data Availability

Not applicable.

## Abbreviations

MSC: Mesenchymal stem cell
SRAS-CoV-2: Severe acute respiratory syndrome coronavirus 2
COVID-19: Coronavirus disease 2019
iRNA: Interfering RNA
miRNAs: MicroRNAs
siRNAs: Small interfering RNAs
PRRs: Pattern recognition receptors
DC: Dendritic cell
ncRNAs: Including non-coding RNAs
HAF: Human amniotic fluid
RNAi: RNA interference
hATTR: Hereditary transthyretin-mediated amyloidosis
S: Spike protein
ACE-2: Angiotensin-converting enzyme 2
TMPRSS2: Transmembrane serine protease 2
ORF: Open reading frame
E: Envelop protein
N: Nucleocapsid protein
M: Matrix protein
NSP: Non-structural protein
RdRp: RNA-dependent RNA polymerase
ATR1: Type 1 Angiotensin II Receptor
PC: Protein convertase
PACE: Paired basic amino acid cleaving enzyme
shRNAs: Short hairpin RNAs
TLRs: Toll-Like Receptors
CRP: C-reactive protein
Cox-2: Cyclooxygenase-2
DAMP: Damage-associated molecular pattern
PAMP: Pathogen-associated molecular patterns
NLRs: Nod like receptors
NF-κB: Nuclear factor kappa B
MAPK: Mitogen-activated protein kinase
JAK–STAT: Janus kinase–signal transducer and activator of transcription
HMGB1: High mobility group box protein 1
GPx: Glutathione peroxidase-1
NOX: NADPH oxidases
iNOS: Inducible nitric oxide synthase
IL-1: Interleukin 1
TNF-α: Tumor necrosis factor alpha
ARDS: Acute respiratory distress syndrome
IL-1β: Interleukin 1 beta
RSV: Respiratory syncytial virus
CRS: Cytokine release syndrome
SOD: Superoxide dismutase
RISC: RNA-induced silencing complex
